# Specific and Rapid Detection of *Mycobacterium tuberculosis* Complex in Clinical Samples by Polymerase Chain Reaction

**DOI:** 10.1155/2012/654694

**Published:** 2012-10-09

**Authors:** Anamika Singh, Vijendra Kumar Kashyap

**Affiliations:** ^1^National DNA Analysis Center, Central Forensic Science Laboratory, 30 Gorachand Road, Kolkata 700014, India; ^2^Sol Sherry Thrombosis Research Center, Temple University School of Medicine, 3400 N. Broad Street, Philadelphia, PA 19140, USA; ^3^Directorate of Forensic Science, MHA, Government of India, CGO Complex, New Delhi 110 003, India

## Abstract

*Background*. Tuberculosis, a global health problem and highly prevalent in India, has always been a serious problem with respect to definitive diagnosis. Polymerase chain reaction (PCR) techniques are now widely used for early detection and species differentiation of mycobacteria, but mostly with their own limitations. We aim to detect and differentiate *Mycobacterium tuberculosis (Mtb)* infections by choosing appropriate target sequences, ideally present in all mycobacterial species (*MTB* complex) and absent in others. *Methods*. Amplification of three target sequences from unrelated genes, namely, *hsp 65* (165 bp), *dnaJ* (365 bp), and insertion element *IS 6110* (541 bp) by PCR was carried out in clinical samples from suspected cases of tuberculosis/ mycobacterioses and healthy controls. *Results*. The sensitivity of this method ranged from 73.33% to 84.61%, and the specificity was 80%. The PCR method was significantly better (*P* = 0.03 and *P* = 0.009) than both smear and culture methods. *Conclusion*. Our trimarker-based PCR method could specifically detect *M. tuberculosis* and *MTB* complex infection from that of major pathogenic NTM and nonpathogenic mycobacteria. This method, by well distinguishing between *MTB* complex and NTM, presented a fast and accurate method to detect and diagnose mycobacterial infections more efficiently and could thereby help in better patient management particularly considering the increase in mycobacterial infections due to emergence of NTM over the past decades.

## 1. Introduction

Tuberculosis is a major public health problem with a total of 9.2 million new cases and 1.7 million deaths from tuberculosis (TB) in 2006. India accounts for one-fifth of the global TB burden (WHO 2008), which has been on the rise due to multidrug-resistant and highly virulent strains of *Mycobacterium tuberculosis* (*Mtb*) [1] and combined effect of HIV. An accurate diagnosis of tuberculosis is desirable before the start of anti-tuberculosis therapy [2]. The laboratory diagnosis of *Mtb* depending on acid-fast bacillus (AFB) smear can yield a result within 24 h. However, smear is not very specific for *Mtb* and also requires 10^3^ to 10^4^ organisms per mL of sputum. Bacterial culture is superior to AFB smear, both in terms of sensitivity and specificity. But, since mycobacteria have very strict growth requirements, culture-based diagnostic methods are slow. Further, diagnoses involving radiological examinations and Tuberculin test help to detect the disease to some extent, but are not very reliable in case of extrapulmonary tuberculosis. The BACTEC system however, gives a very quick result compared to culture, but it is expensive and involves handling of radioisotopes (BACTEC 460). The fluorometric BACTEC MGIT 960 is fast, efficient, and sensitive but expensive to be used in poor economies. In view of these, accurate and early diagnosis of tuberculosis (TB) is a critical step in the management and control of TB. Polymerase chain reaction has so far been a very useful tool for rapid detection of mycobacterial DNA in clinical specimens such as sputum, bronchial lavage, cerebrospinal fluid (CSF), and ascitic fluid. However, in-house tests vary widely in their accuracy and factors that contribute to heterogeneity in test accuracy, are not well characterized. Definitive diagnosis of tuberculosis and mycobacterioses (nontuberculous mycobacteria (NTM) infections) has always been a serious problem. These methods also have their own limitations due to region-specific variations in the genome of mycobacteria [3–5]. Our study describes a PCR assay using *MTB*-complex-specific DNA markers encompassing 165, 365, and 541 bp target fragments of three unrelated genes, namely, *hsp 65, dnaJ* and insertion element *IS 6110*, respectively, of *M. tuberculosis* in tuberculosis suspected patients from Kolkata, India. Considering the increase in mycobacterial infections resulting from the emergence of NTM over the past decades, there is also need for a method that can detect virtually any deep-seated mycobacterial organism present in small numbers in suspected clinical samples irrespective of the mycobacterial species affiliation. 

## 2. Materials and Methods

### 2.1. Subjects

The study samples were suspected cases of pulmonary tuberculosis visiting the Chest Outpatient Department of Calcutta National Medical College and Hospital (CNMCH). This study was approved by the Ethical Committees of the institutions involved, and all the subjects signed informed consent documents before entering into this study. A total of 60 subjects, including suspected cases and healthy controls were studied. The suspected TB cases were further categorized into three groups (Group I–III) based on laboratory diagnostic results (AFB-smear, culture and antero-posterior chest X-ray), clinical symptoms, and past history. The clinical and demographic data on studied subjects is presented in Table 1. Five to ten mL of early morning sputum samples were collected from all the subjects. All the samples were smeared and screened with conventional microbiological test such as Ziehl-Neelsen acid fast staining for recording smear positivity and further cultured on Lowenstein-Jensen slants according to standard method. Group I (*n* = 26) had cases, which were positive by smear Ziehl-Neelsen staining and also culture positive, while Group II (*n* = 15) had smear negative but culture and radiologically positive samples. In some patients (*n* = 9), the laboratory diagnosis for *Mtb* infection was negative but based on their clinical symptoms, they were considered as having other respiratory diseases (than PTB) or mycobacterioses (Group III). However, these patients did not have any past history of tuberculosis and subsequent treatment. The healthy control subjects (designated as Gr IV, *n* = 10) were with no clinical symptoms or history and were diagnosed negative for *Mtb* infection (bacteriologically negative). All the 60 samples were further analyzed by PCR method. 

### 2.2. Isolation of DNA from Sputum Samples

The sputum samples were decontaminated followed by DNA isolation according to the previously published method [6].

### 2.3. PCR Amplification

**Figure 1:**
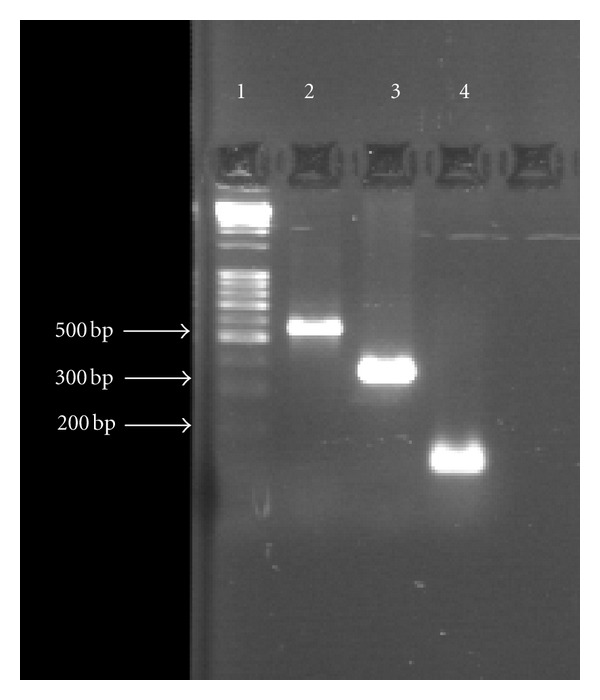
PCR showing 165, 365, and 541 bp fragment of *M. tuberculosis *reference strain H37Rv (positive control). Lane 1: gene marker, Lane 2: 541 bp amplicon, Lane 3: 365 bp amplicon, and Lane 4: 165 bp amplicon.

**Figure 2:**
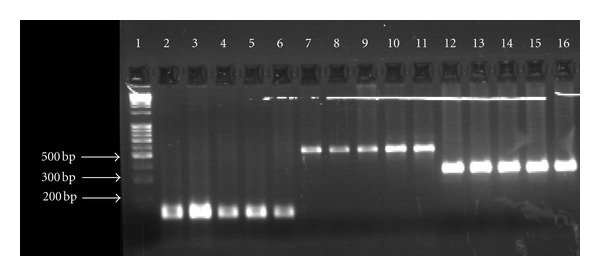
PCR showing 165, 365, and 541 bp target sequence amplified in case samples. Lane 1- gene marker, Lanes: 2–6, 165 bp amplicon, Lanes: 7–11; 541 bp amplicon, Lanes: 12–16, 365 bp amplicon.

The PCR amplification targeted two conserved regions and one insertion element of mycobacterial genome. A 165 bp conserved region of a gene coding for 65 k Dalton antigen protein of *M. tuberculosis* was amplified using primer set published previously [7]. Similarly, a 365 bp region (between sequence position 1377–1741) of *dnaJ* gene of *M. tuberculosis* was amplified using genus-specific primers described earlier by Takewaki et al. (1993) [8]. The 541 bp region within insertion element IS6110 of *M. tuberculosis* was amplified using oligonucleotides designated as Pt-8 and Pt-9 by Kox et al. 1994 [9]. Ten microliters of the amplified PCR product were fractionated electrophoretically on a 2% agarose gel, stained with ethidium bromide and visualized under UV-transilluminator for the accuracy and specificity of PCR amplification, using PhiX174 HaeIII digest as gene ruler. All the PCR amplifications were subjected to internal laboratory standardization using template DNA from *M. tuberculosis* reference strain H37Rv as positive control (Figure 1). Briefly, 50–100 ng of purified total cellular DNA was amplified with thermostable Taq DNA polymerase in Gene Amp PCR System 9700 (Applied Biosystems, Foster City, CA). To establish positivity, we added 50 uL of reaction mixture containing 10 mm Tris-HCl (pH 8.3), 50 mm KCl, 1.5–2.5 mm MgCl_2_, 0.01% (w/v) gelatin, 20–50 pmol of respective primers (described earlier), 2.0–2.5 nmol of each of the four deoxynucleoside triphosphates (dATP, dCTP, dGTP and dTTP), 1 U of Taq DNA polymerase (Applied Biosystems, Foster City, CA). The PCR cycle conditions were slight modifications of the previously published one for 165 bp hsp gene fragment (94°C for 20 s to denature the DNA, then cooled to 63°C for 20 s, heating to 72°C for 1 min for extension, cycle repeated 30 times with final incubation at 72°C for 10 min), for 365 bp *dnaJ* gene fragment (94°C for 30 s to denature the DNA, then cooled to 65°C for 60 s, heating to 72°C for 2.5 min for extension, cycle repeated 35 times with final incubation at 72°C for 10 min) and for 541 bp IS6110 sequence (94°C for 60 s to denature the DNA, then cooled to 65°C for 1.5 min, heating to 72°C for 3.5 min for extension, cycle repeated 38 times with final incubation at 72°C for 10 min) of mycobacterial DNA.  Figure 2 shows the amplification of all the above mentioned genetic markers in clinical samples.

### 2.4. Statistical Analysis

The sensitivity, specificity, positive predictive value (PPV), and negative predictive value (NPV) and Chi-square tests were calculated using ACASTAT Software [10]. The results of 2 × 2 Chi-square tests and *P* values were uncorrected ones, unless otherwise indicated. A *P* value ≤0.05 was considered statistically significant. 

## 3. Results 

Out of 60 studied samples, 26 were positive by smear Ziehl-Neelsen staining and were also culture positive (Gr I). We detected 22 samples of Gr I as positive by PCR (84.62%). Out of 15 samples of Group II (smear negative but culture and radiologically positive), 11 samples (73.33%) turned out positive by PCR method. Hence, our trimarker system-based polymerase chain reaction detected 33 (80.5%) positive out of 41 patients (combined Gr I & II). We also found 7 samples (77.78%) out of 9 from the III^*rd*⁡^ Group as positive for 365 bp *dnaJ* gene (but not for rest two genes) indicating that they might be cases with mycobacterioses but not tuberculosis. In the controls (Gr IV, *n* = 10), none was PCR positive for any of the 3 markers used in this study. However, there were 4 samples in Gr I and 2 in Gr II, which could not be amplified for *IS6110,* possibly due to the presence of PCR inhibitors. Similarly, 2 samples in Gr II did not amplify for *hsp 65* gene marker. The PCR amplification results for Gr III clearly demonstrated infection with nontuberculous mycobacteria. The specificity of this study was 80%, and sensitivity range was 73.33% (Gr.II) to 84.61% (Gr.I). Also, the PPV and NPV were 77.78–91.67% and 66.67–80%, respectively. When compared to the smear and culture methods, the results were statistically significant (*P* = 0.030 and *P* = 0.009, resp.), showing that the PCR technique had a significantly higher ability to detect tuberculosis caused by *Mycobacterium tuberculosis* complex. The results of this study are presented in Tables 2, 3, and 4.

## 4. Discussion 

Pulmonary tuberculosis is one of the highly infectious diseases and mostly prevalent in India, but it still bears with it the problem of definitive diagnosis. PCR amplification of mycobacterial DNA is a highly sensitive and specific technique to detect Tuberculosis. This also performs better than smear (which needs >10,000 bacilli/mL sample for detection) as well as culture method (which has restricted growth conditions). In these cases, PCR helps to diagnose whether the positivity (smear or culture) is due to *MTB* complex bacteria or other nontuberculous infection. In case of negative Ziehl-Neelsen staining, PCR helps to confirm the diagnosis. Our Data clearly showed that there is a significant difference between smear and PCR (*P* = 0.030) and between culture and PCR (*P* = 0.009) methods, further signifying that the PCR method has proved better than smear and culture methods in this study. However, many of the mycobacterial PCR assays employing species-specific primers allow for the detection of a single or limited number of mycobacterial species [11–16]. The efficiency and success of any rapid diagnostic PCR method depend on the appropriate DNA target sequences chosen for amplification. In this study, the marker system chosen is ideally applicable to most of the mycobacterial species (*M. tuberculosis, M. bovis, M. bovis BCG, M. africanum, and M. microti*) of *MTB* complex. The Success of this method for the detection and identification of mycobacteria further increased due to inclusion of one target sequence (*dnaJ* gene) for NTM. A meta-analysis published recently [17] demonstrated that use of *IS6110* as a target of amplification was significantly associated with increased accuracy of the PCR-based detection test. However, although this sequence is present in multiple copies in the bacterial genome, some strains from certain parts of the world lack it [18, 19], and this may cause false-negative results further affecting the sensitivity of the assay. A possible solution to this problem would be to amplify more than one target sequences. Hence, we have also used 165 and 365 bp target DNA sequences in our study. The 365 bp *dnaJ *gene marker is a genus-specific, one which has a broader spectrum of detection and can be amplified from all mycobacterial (including nontuberculous and non-pathogenic) species, while the 541 bp *IS6110* and 165 bp 65 kDa antigen protein gene sequences are amplifiable from almost all the mycobacterial species of *MTB* complex (but not from NTM) [7, 9], thus accounting for their higher sensitivity. So far no single target sequence has provided 100% sensitivity and a total absence of false positive when used alone. It was also observed that multiplex PCR did not contribute to increase the diagnostic accuracy in a large meta-analysis study [17]. Our trimarker based PCR method could sensitively and specifically detect clinically important mycobacterial infections such as those of *M. tuberculosis*, *M. tuberculosis* complex other than *M. tuberculosis* (especially *M. bovis*) and differentiate from major pathogenic NTM and nonpathogenic mycobacteria. Hence, this method presented a fast and efficient technique to diagnose tuberculosis infection and differentiate it from other mycobacterial infections in order to help better patient management. However, since some samples could not be amplified properly for certain DNA sequences in this study, it is important to consider PCR inhibitors and the sensitivity of the assay could further improve with effective control of such inhibitors.

## 5. Conclusions

The study presents a trimarker based PCR technique, which also applies to a wide variety of clinical samples and hence evaluated as a useful technique in the diagnosis of pulmonary tuberculosis. This method is very useful in cases where Ziehl-Neelsen staining and/or culture results are negative. By including one relatively less-specific marker (365 bp *dnaJ* gene), this PCR method also facilitates discrimination of *MTB* complex-specific infection from nontuberculous or nonpathogenic mycobacterial infections. The fact that DNA amplification can detect mycobacterial DNA sequences in the presence of excess amounts of human DNA makes it especially useful when quick results are required in certain clinical circumstances. This may also be useful when large-scale screening of mycobacteria is required, such as in parts of the countries where tuberculosis is still endemic and remains a major public health problem. 

## Figures and Tables

**Table 1:** Characteristics of clinical samples and controls based on conventional diagnostic methods and other clinical symptoms.

Category	Status of the samples				Samples (*n*)	Age (Range)	Sex (Male/Female) (*n*)
	*AFB staining (Z-N)^#^	Culture	X-ray	Other			
Group I	+	+	+	+	26	14–72	19/7
Group II	−	+	+	+	15	14–65	10/5
Group III	−	−	−	+	9	14–75	4/5
Group IV	−	−	−	−	10	15–60	8/2

*AFB: acid fast bacilli; ^#^Z-N: ziehl-neelsen; +: positive; −: negative.

**Table 2:** Detection of mycobacterial infection by polymerase chain reaction.

Category	Samples (*n*)	PCR results					No PCR- amplification		*dna*J
		Positive *n* (%)	Sensitivity (%)	Specificity (%)	PPV (%)	NPV (%)	For (*n* [%])		
							*IS6110*	*hsp *65	
Group I	26	22 (84.62)*	84.6	80	91.67	66.67	4 (15.38)		
Group II	15	11 (73.33)*	73.33	80	84.62	66.67	2 (13.33)	2 (13.33)	
Group III	9	7 (77.78)^ #^	77.78	80	77.78	80			2 (22.22)
Group IV	10	0 (0)	—	—	—	—	10 (100)	10 (100)	10 (100)

*Indicates positive result for all three markers; ^#^indicates positive result for only *dna*J; PPV: positive predictive value; NPV: negative predictive value.

**Table 3:** Comparison of PCR and smear methods for detection of mycobacterial infections.

Samples	Smear *n* (%)	PCR *n* (%)	*P*
Positive	26 (43.33)	42 (70)	0.030*
Negative	34 (56.66)	18 (30)	

*P value < 0.05.

**Table 4:** Comparison of PCR and culture methods for detection of mycobacterial infections.

Samples	Culture *n* (%)	PCR *n* (%)	*P*
Positive	41 (68.33)	42 (70)	0.009*
Negative	19 (31.67)	18 (30)	

*P value < 0.05.

## References

[3] O’Shea B, Khare S, Bliss K (2004). Amplified fragment length polymorphism reveals genomic variability among *Mycobacterium avium* subsp. *paratuberculosis* isolates. *Journal of Clinical Microbiology*.

[4] Weil A, Plikaytis BB, Butler WR, Woodley CL, Shinnick TM (1996). The mtp40 gene is not present in all strains of *Mycobacterium tuberculosis*. *Journal of Clinical Microbiology*.

[5] Homolka S, Niemann S, Russell DG, Rohde KH (2010). Functional genetic diversity among *Mycobacterium tuberculosis* complex clinical isolates: delineation of conserved core and lineage-specific transcriptomes during intracellular survival. *PLoS Pathogens*.

[6] Peres RL, Maciel ELN, Morais CG (2009). Comparison of two concentrations of NALC-NaOH for decontamination of sputum for mycobacterial culture. *International Journal of Tuberculosis and Lung Disease*.

[7] Pao CC, Yen TSB, You JB, Maa JS, Fiss EH, Chang CH (1990). Detection and identification of *Mycobacterium tuberculosis* by DNA amplification. *Journal of Clinical Microbiology*.

[8] Takewaki SI, Okuzumi K, Ishiko H, Nakahara KI, Ohkubo A, Nagai R (1993). Genus-specific polymerase chain reaction for the mycobacterial dnaJ gene and species-specific oligonucleotide probes. *Journal of Clinical Microbiology*.

[9] Kox LFF, Rhienthong D, Miranda AM (1994). A more reliable PCR for detection of *Mycobacterium tuberculosis* in clinical samples. *Journal of Clinical Microbiology*.

[10] AcaStat Software http://www.acastat.com/.

[11] Del Portillo P, Murillo LA, Patarroyo ME (1991). Amplification of a species-specific DNA fragment of *Mycobacterium tuberculosis* and its possible use in diagnosis. *Journal of Clinical Microbiology*.

[12] Park H, Jang H, Kim C (2000). Detection and identification of mycobacteria by amplification of the internal transcribed spacer regions with genus- and species-specific PCR primers. *Journal of Clinical Microbiology*.

[13] Obieta MPP, Ang CF, Obieta MYP (2008). Evaluation of a PCR amplification method for detection of mycobacterial DNA in formalin-fixed paraffin embedded skin tissues. *Philippine Journal of Microbiology and Infectious Diseases*.

[14] Lachnik J, Ackermann B, Bohrssen A (2002). Rapid-cycle PCR and fluorimetry for detection of mycobacteria. *Journal of Clinical Microbiology*.

[15] Hermans PMW, Schuitema ARJ, Van Soolingen D (1990). Specific detection of *Mycobacterium tuberculosis* complex strains by polymerase chain reaction. *Journal of Clinical Microbiology*.

[16] Williams KJ, Ling CL, Jenkins C, Gillespie SH, McHugh TD (2007). A paradigm for the molecular identification of Mycobacterium species in a routine diagnostic laboratory. *Journal of Medical Microbiology*.

[17] Flores LL, Pai M, Colford JM, Riley LW (2005). In-house nucleic acid amplification tests for the detection of *Mycobacterium tuberculosis* in sputum specimens: meta-analysis and meta-regression. *BMC Microbiology*.

[18] Das S, Paramasivan CN, Lowrie DB, Prabhakar R, Narayanan PR (1995). *IS6110* restriction fragment length polymorphism typing of clinical isolates of *Mycobacterium tuberculosis* from patients with pulmonary tuberculosis in Madras, South India. *Tubercle and Lung Disease*.

[19] Lok KH, Benjamin WH, Kimerling ME (2002). Molecular differentiation of *Mycobacterium tuberculosis* strains without *IS6110* insertions. *Emerging Infectious Diseases*.

